# Optimizing the anesthetic management of factor XI deficiency in obstetrics: A case report using ROTEM

**DOI:** 10.1016/j.ijscr.2025.111566

**Published:** 2025-06-26

**Authors:** Asma Ladib, Jihen Ladib, Fethi Jebali, Asma Korbi, Imen Ghaddab, Mouna Sassi

**Affiliations:** a Department of Anesthesia and Intensive Care B, Center of Maternity and Neonatology of Monastir (CMNM), Fattouma Bourguiba University Hospital, Monastir, Tunisia; b Department of Pharmacy, Fattouma Bourguiba University Hospital, Monastir, Tunisia; c Department of Gynecology and Obstetrics, Center of Maternity and Neonatology of Monastir (CMNM), Fattouma Bourguiba University Hospital, Monastir, Tunisia; d Laboratory of Hematology, Center of Maternity and Neonatology of Monastir (CMNM), Fattouma Bourguiba University Hospital, Monastir, Tunisia

**Keywords:** Case report, Factor XI deficiency, ROTEM, Cesarean section, Regional anesthesia, Tranexamic acid

## Abstract

**Introduction:**

Factor XI (FXI) deficiency is a rare coagulation disorder with poor correlation between FXI levels and bleeding risk, posing challenges in obstetric anesthesia. Neuraxial anesthesia is often avoided due to theoretical concerns about spinal hematoma. However, viscoelastic testing such as rotational thromboelastometry (ROTEM) may offer functional insights into coagulation status.

**Case presentation:**

We report the case of a 28-year-old primigravid woman with an incidentally discovered moderate FXI deficiency (52.6 %) during routine preoperative evaluation for suspected macrosomia. The patient had a minimal bleeding history (ISTH-BAT score: 1). ROTEM (INTEM assay) showed mildly prolonged clotting time (CT: 236 s) and slightly reduced early clot firmness (A5: 42 mm), with preserved maximum clot firmness (MCF: 68 mm). Given the integrative evaluation—moderate FXI deficiency, reassuring ROTEM profile, and absence of bleeding symptoms—spinal anesthesia was safely performed under prophylactic tranexamic acid. The elective Cesarean section was uneventful, with estimated blood loss of 450 mL and no hemostatic complications.

**Discussion:**

This case illustrates how ROTEM, although not diagnostic alone, can support multidisciplinary decision-making in obstetric anesthesia for FXI-deficient patients. The postoperative ROTEM improvement was likely multifactorial, including postpartum procoagulant shift and TXA use, rather than a direct effect of ROTEM-guided intervention.

**Conclusion:**

ROTEM may serve as a useful adjunct in the individualized management of FXI deficiency during pregnancy. However, its role remains complementary, and further prospective studies are needed to clarify its predictive value and establish standardized thresholds.

## Highlights

• FXI deficiency in pregnancy presents unique anesthetic challenges due to unpredictable bleeding risk.
• Viscoelastic testing (ROTEM) revealed mild abnormalities but preserved clot stability preoperatively.
• ROTEM supported a conservative approach, avoiding FXI replacement in a parturient undergoing Cesarean section.
• Tranexamic acid was used prophylactically; ROTEM follow-up confirmed effective postpartum hemostasis.
• ROTEM may serve as an adjunctive tool for personalized anesthetic decisions in FXI-deficient pregnancies.

## 1 Introduction

Hemostatic disorders in obstetrics pose a major challenge in anesthesia, requiring a delicate balance between preventing hemorrhagic risk and avoiding thromboembolic complications. Factor XI (FXI) deficiency, also known as hemophilia C (1), is a rare coagulation disorder characterized by unpredictable clinical expression and a weak correlation between plasma FXI levels and actual hemorrhagic risk (2). Unlike other coagulopathies, this deficiency is often incidentally discovered during preoperative screening, making perioperative management particularly complex, especially in obstetric anesthesia (3).

The assessment of hemorrhagic risk has historically relied on conventional coagulation tests, such as activated partial thromboplastin time (aPTT), which are often insufficient to predict clinical bleeding. Recently, global hemostasis tests, particularly rotational thromboelastometry (ROTEM), have emerged as promising tools for a more refined functional evaluation of coagulation (4). By providing a dynamic analysis of clot formation and stability, ROTEM could play a key role in optimizing anesthetic and hemostatic strategies for FXI-deficient patients.

We report the case of a primigravid woman in whom an isolated prolongation of activated partial thromboplastin time (aPTT) using kaolin as activator (aPTT kaolin-activated) led to the discovery of moderate FXI deficiency (52.6 %).Through this observation, we analyze the diagnostic and therapeutic implications of FXI deficiency in obstetrics, with a focus on the role of ROTEM in anesthetic and hemostatic decision-making, as well as the most appropriate anesthetic technique.

This case report has been reported in line with the SCARE 2025 criteria (5).

## 2 Presentation of case

A 28-year-old primigravid patient, M.C., was followed for an uneventful pregnancy by a private gynecologist. During the third-trimester consultation at 38 weeks of gestation, a coagulation assessment, including a complete blood count, prothrombin time (PT), and aPTT (kaolin-activated), was requested as part of delivery preparation due to suspected fetal macrosomia, raising the possibility of an elective cesarean section.

Biological investigations revealed an isolated and incidentally discovered prolongation of the aPTT (kaolin-activated) (Table 1).

**Table 1:** Initial hemostasis assessment.

Parameter	Result	Normal values
Complete blood count		
Hemoglobin (g/dL)	13.8	12–16
Platelets (G/L)	152	150–400
Coagulation		
Prothrombin time (PT, %)	90	70–120
aPTT (silica-activated) (s)	34.4/31 (ratio 1.10)	28–35
aPTT (kaolin-activated) (s)	41.3/31 (ratio 1.33)	28–36
aPTT (ellagic acid-activated)(s)	37/26 (ratio 1.42)	28–36
Fibrinogen (g/L)	5.25	2–4
Coagulation factors		
Factor VIII (%)	156.5	50–150
von Willebrand Factor (VWF:Rco, %)	123.1	50–160
Factor IX (%)	120	60–140
Factor XI (%)	52.6	70–150
Factor XII (%)	136.8	50–150

Activated partial thromboplastin time (aPTT) was assessed using different contact activators (kaolin, ellagic acid, and silica), as these reagents have variable sensitivity to intrinsic pathway abnormalities. The observed discrepancies—namely, the prolonged aPTT (kaolin-activated) and aPTT (ellagic acid-activated) versus the near-normal aPTT (silica-activated)—reflect these reagent-specific differences and supported the diagnosis of mild FXI deficiency (52.6 %), which was subsequently confirmed by dilutional studies.

Further history-taking uncovered previously unexplored menorrhagia. The clinical assessment revealed no other bleeding symptoms such as epistaxis, gingival bleeding, easy bruising, or postoperative bleeding, and no family history of hemorrhagic disorders. The ISTH-BAT score was estimated at 1. Hematologic evaluation confirmed a moderate factor XI (FXI) deficiency (52.6 %) with preserved dilution parallelism, accounting for the prolonged aPTT (kaolin-activated). Based on this limited bleeding phenotype and the laboratory findings, the multidisciplinary assessment concluded a low hemorrhagic risk. The hematology team recommended increased vigilance during the peripartum period, with the possible use of fresh frozen plasma (FFP) if clinically indicated, and prophylactic antifibrinolytic therapy in light of the association between FXI deficiency and hyperfibrinolysis.

At 40 weeks and 3 days of gestation, the patient was scheduled for an elective cesarean section due to fetal macrosomia.

In the operating room, the patient was positioned and monitored via electrocardiography, non-invasive blood pressure, and pulse oximetry. A preoperative laboratory assessment confirmed no worsening of the coagulation disorder. ROTEM analysis was performed for a more detailed evaluation of the hemostatic profile.

**Fig. 1:**
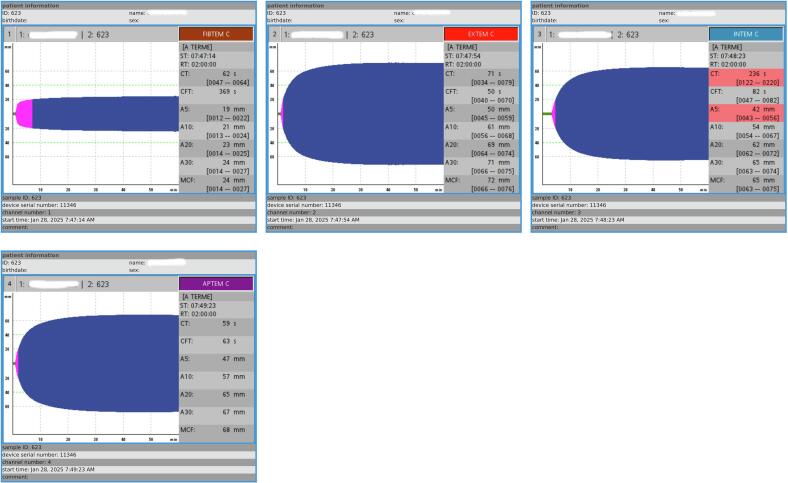
Rotational thromboelastometry (ROTEM) traces of the clinical case – Preoperative ROTEM. APTEM: anti-fibrinolysis; EXTEM: extrinsic coagulation pathway; FIBTEM: fibrinogen contribution to clot firmness; INTEM: ellagic acid-activated intrinsic pathway. ROTEM parameters: CT (clotting time, [s]); CFT (clot formation time, [s]); MCF (maximum clot firmness, [mm]); A5, A10 (amplitudes at 5 and 10 min after CT, [mm]). Reference ranges correspond to institutional values routinely used at our hospital (based on internal validation).

The ROTEM results revealed two abnormalities on the INTEM assay: a prolonged coagulation time (CT) of 236 s (reference range: 122–220 s), indicating delayed clot initiation, and a slightly reduced amplitude at 5 min (A5) of 42 mm (reference range: 43–56 mm), suggesting relative hypocoagulability during the early phase of clot polymerization. However, subsequent values showed progressive clot strengthening with an A10 of 54 mm, A20 of 62 mm, and A30 of 65 mm (all within reference ranges), indicating overall preserved clot firmness and stable hemostatic function. The Maximum Clot Firmness (MCF), which reflects the final mechanical strength of the clot and thus the overall hemostatic potential, was 68 mm on the preoperative INTEM, confirming preserved clot firmness (Fig. 1, Table 2).

**Table 2:** Institutional Reference Ranges for ROTEM Parameters in Pregnant Women.

Assay	Parameter	Reference range
FIBTEM	CT (s)	47–64
	A5 (mm)	12–22
	A10 (mm)	13–24
	A20 (mm)	14–25
	A30 (mm)	14–27
	MCF (mm)	14–27
EXTEM	CT (s)	34–79
	CFT (s)	40–70
	A5 (mm)	45–59
	A10 (mm)	56–68
	A20 (mm)	64–74
	A30 (mm)	66–75
	MCF (mm)	66–76
INTEM	CT (s)	122–220
	CFT (s)	47–82
	A5 (mm)	43–56
	A10 (mm)	54–67
	A20 (mm)	62–72
	A30 (mm)	63–74
	MCF (mm)	63–75

Considering the moderate FXI deficiency, the absence of any bleeding history, and the overall preserved hemostatic potential confirmed by ROTEM, spinal anesthesia was judged to be an appropriate and safe option.

To prevent hemorrhagic risk, 1 g of tranexamic acid was administered 30 min before surgery, followed by an additional 1 g intraoperative maintenance dose. Spinal anesthesia was performed with 10 mg hyperbaric bupivacaine, 100 μg morphine, and 3 μg sufentanil, achieving a sensory level at T4.

Fetal extraction resulted in the delivery of a male neonate with an Apgar score of 9/10 and a birth weight of 4 kg. The procedure lasted 55 min and was uneventful. The estimated blood loss (EBL) during the Cesarean section was approximately 450 mL, which falls within the expected range for an uncomplicated elective Cesarean delivery.

**Fig. 2:**
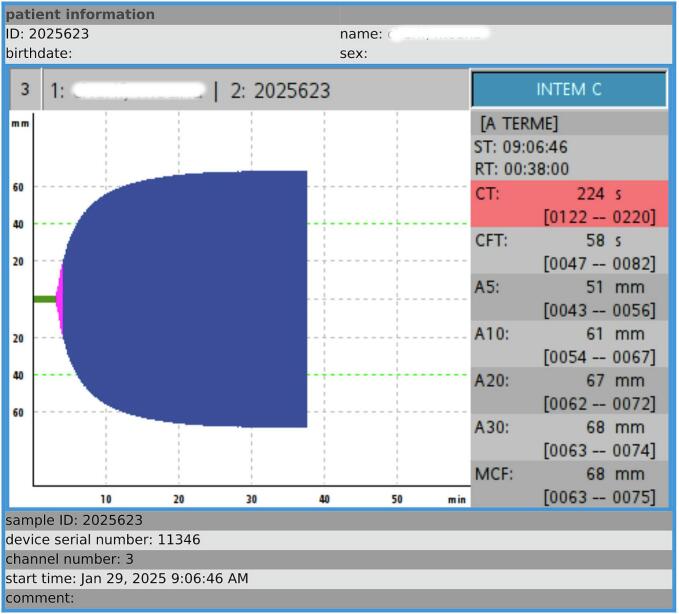
Rotational Thromboelastometry (ROTEM) Traces of the Clinical Case – Postoperative ROTEM (INTEM Module). INTEM: ellagic acid activated intrinsic pathway (CT, clotting time[s]; CFT, clot formation time[s]; MCF, maximum clot firmness [mm]; A5, amplitude time point of 5 min after CT [mm]; A10, amplitude time point of 10 min after CT [mm];

The patient was transferred to the ICU for postoperative monitoring. A ROTEM follow-up performed on postoperative day 1 (J1) showed further improvement in coagulation parameters, with normalization of A5, a reduction in CT, and a MCF of 66 mm, consistent with sustained adequate clot strength over time (Table 3, Fig. 2).

**Table 3:** Postoperative hemostasis assessment (J1).

Parameter	Result	Normal values
Complete blood count		
Hemoglobin (g/dL)	11.8	12–16
Platelets (G/L)	127	150–400
Coagulation		
Prothrombin Time (PT, %)	90	70–120
Kaolin cephalin clotting time (KCCT, s)	41/31	28–36
Fibrinogen (g/L)	4.8	2–4

Thromboprophylaxis with enoxaparin (4000 IU/day) was initiated on J1. The postoperative course was favorable, allowing transfer to a conventional unit after 48 h. The patient was discharged on postpartum day 4 with Microval contraception and an outpatient follow-up appointment.

## 3 Discussion

Factor XI (FXI) deficiency, also known as hemophilia C, is a rare coagulation disorder characterized by a heterogeneous clinical expression, making the stratification of hemorrhagic risk complex. Unlike hemophilia A and B, plasma FXI levels do not strictly correlate with bleeding risk, complicating anesthetic management in obstetric settings (6).

1. ROTEM: a key decision-making tool for a personalized approach

The anesthetic management of patients with FXI deficiency has historically relied on conventional coagulation tests, particularly activated partial thromboplastin time (aPTT) and FXI activity levels. However, these parameters remain poorly predictive of bleeding phenotype, which is highly variable and not linearly correlated with FXI concentrations (7). In this context, rotational thromboelastometry (ROTEM) offers a dynamic, functional, and real-time assessment of the coagulation process, thereby providing additional insight into the patient's true hemostatic capacity (4).

In our case, the FXI activity was 52.6 %, a level generally considered sufficient to support safe neuraxial anesthesia in the absence of personal or familial bleeding history. Nevertheless, we elected to complement this biological and clinical information with ROTEM analysis to guide individualized perioperative risk assessment.

Preoperative ROTEM revealed two mild abnormalities in the INTEM assay: a slightly prolonged coagulation time (CT) of 236 s (normal range: 122–220 s), indicating delayed initiation of clotting, and a modest reduction in amplitude at 5 min (A5) to 42 mm (normal: 43–56 mm), suggestive of transient hypocoagulability during early clot formation. However, clot firmness improved rapidly over time with amplitudes of 54 mm at 10 min (A10), 62 mm at 20 min (A20), and a maximum clot firmness (MCF) of 65 mm, reflecting effective clot propagation and stability. These findings point to preserved thrombin generation and intact downstream coagulation mechanisms, likely via direct factor X activation (8).

Rather than relying solely on the FXI level, we interpreted ROTEM results within the broader clinical context. The integration of FXI activity, clinical bleeding history, and ROTEM parameters enabled a risk-stratified and conservative anesthetic strategy. Specifically, ROTEM findings reassured us that additional prohemostatic interventions such as fresh frozen plasma (FFP) or FXI concentrate were unnecessary. The decision to proceed with spinal anesthesia was supported by this composite assessment and reinforced by prophylactic administration of tranexamic acid (1 g before and 1 g during surgery), mitigating fibrinolytic activity and reducing bleeding risk without promoting thrombosis (9).

This case illustrates the added value of ROTEM as a precision medicine tool, allowing clinicians to move beyond arbitrary FXI thresholds and adopt an individualized approach that prioritizes functional hemostatic capacity, clinical phenotype, and real-time risk evaluation.

2. Surgical context and hemostatic risk in FXI deficiency

The type of surgical procedure, particularly Cesarean section versus vaginal delivery, is an important determinant of bleeding risk in obstetric patients with FXI deficiency. Cesarean delivery is generally associated with greater surgical trauma, higher vascular disruption, and increased median blood loss (typically 700–1000 mL vs. 300–500 mL for vaginal delivery in the general population). These factors may heighten bleeding risk, especially in women with underlying coagulopathies.

However, current literature indicates that in parturients with FXI deficiency, the risk of significant bleeding is not solely determined by the mode of delivery. Evidence suggests that factors such as baseline FXI activity and individual bleeding history are more predictive of hemorrhagic outcomes than the surgical context alone. In particular, Cesarean section, although associated with greater surgical trauma and blood loss in the general population, does not necessarily confer an increased risk of postpartum hemorrhage in this specific population when appropriate perioperative management is implemented (10).

In our case, the patient underwent an elective Cesarean section with a moderate FXI deficiency (52.6 %) and a reassuring ROTEM profile. Estimated blood loss was 450 mL—within expected limits for this type of surgery—and no bleeding complications occurred. This supports the value of individualized perioperative assessment combining FXI level, bleeding phenotype, and functional hemostasis (via ROTEM), even in higher-risk surgical scenarios.

3. Spinal anesthesia and FXI deficiency: a reevaluation of contraindications?

Spinal anesthesia has long been avoided due to the theoretical risk of epidural hematoma; however, no recent data support this concern when performed in patients with FXI levels ≥30 IU/dL and no major bleeding history (11). A large retrospective study analyzing 199 neuraxial anesthetics in FXI-deficient patients reported no hemorrhagic complications (12).

In our case, ROTEM played a central role in validating spinal anesthesia alongside clinical and laboratory assessments. This approach challenges the heterogeneous practices observed in obstetric anesthesia, where spinal anesthesia is still sometimes avoided in FXI-deficient patients despite limited evidence supporting an increased risk of adverse events.

4. Postoperative ROTEM improvement: the impact of tranexamic acid and the postpartum state

The evolution of ROTEM parameters on postoperative day 1 (POD1) showed partial correction of clotting time (CT: from 236 to 224 s, still borderline) and normalization of A5 (from 42 mm to 51 mm). This improvement is likely multifactorial:

• Postpartum procoagulant shift: The immediate postpartum period is characterized by a physiological increase in coagulation factors such as fibrinogen, factor VIII, and von Willebrand factor, which may contribute to spontaneous normalization of viscoelastic parameters, particularly A5 and MCF (13).
• Antifibrinolytic effect of tranexamic acid (TXA): While TXA's primary mechanism is the inhibition of fibrinolysis through plasminogen binding, its administration may have indirectly stabilized early clot formation, contributing to the observed rise in A5. However, in the absence of hyperfibrinolysis (APTEM trace was normal), its impact on CT remains uncertain and likely minimal.
• Resolution of transient peripartum changes: Potential mild hemodilution from intraoperative fluid administration, plasma volume redistribution, or hormonal influences may have altered the initial ROTEM profile. Since the preoperative ROTEM was performed before any IV fluids were administered, hemodilution is unlikely to explain the baseline abnormalities, but may have influenced early postoperative measurements.
• Biological variability: As previously reported [ref], inter-individual and intra-individual variability in ROTEM parameters may also contribute to these observed changes.

Although the respective contribution of each mechanism cannot be definitively isolated in this single-case observation, the improvement in ROTEM parameters, absence of bleeding, and stable hemodynamic status support the conclusion that hemostasis remained effectively preserved.

In parallel, a decrease in hemoglobin from 13.8 to 11.8 g/dL was observed on postoperative day 1, despite an estimated blood loss (EBL) of only 450 mL, which falls within the expected range for an elective Cesarean delivery. This apparent discrepancy is well described in the postpartum context and reflects a combination of physiological factors beyond blood loss alone. In particular, hemodilution secondary to intraoperative fluid administration, plasma volume redistribution, and uterine autotransfusion after placental separation all contribute to transient reductions in hemoglobin levels. The absence of hemodynamic instability and the reassuring ROTEM profile further support the conclusion that effective hemostasis was maintained.

5. Viscoelastic testing in pregnancy with FXI deficiency: insights and gaps

Current literature on the utility of viscoelastic testing (ROTEM/TEG) in pregnant women with FXI deficiency remains limited and heterogeneous. Although ROTEM is sensitive to reduced FXI activity, its correlation with bleeding risk is inconsistent. Wheeler et al. reported that FXI-deficient parturients may present with ROTEM abnormalities such as prolonged clotting times or reduced clot firmness, but these findings are poorly predictive of hemorrhagic outcomes. Notably, no clear therapeutic thresholds have been established for this population (14).

Similarly, the case described by Martinez-Lopez et al. illustrates the use of ROTEM to monitor coagulation status and guide administration of tranexamic acid and plasma. However, the absence of standardized algorithms and the reliance on individual interpretation highlight the current limitations of this strategy in obstetric settings (6).

In our case, the mild preoperative ROTEM anomalies (INTEM CT 236 s, A5 42 mm) were interpreted in context, and their evolution postoperatively supported the absence of a clinically significant coagulopathy. Nonetheless, we acknowledge that ROTEM findings alone cannot be used to infer bleeding risk or dictate management in FXI deficiency during pregnancy, reinforcing the need for integrative, multidisciplinary assessment.

6. Limitations and Future Directions

This case report has inherent limitations. As a single-center observation, our findings cannot establish causality or be generalized to all FXI-deficient parturients. While ROTEM provided valuable individualized insights, its predictive value for bleeding risk in this population requires validation in larger prospective studies. Furthermore, the absence of standardized ROTEM thresholds for obstetric FXI deficiency highlights the need for multicenter collaborations to develop evidence-based guidelines.

7. Toward a revision of current guidelines?

While this single case cannot justify changes to current guidelines, it illustrates how functional hemostatic assessment using ROTEM may complement traditional FXI activity levels and clinical history in guiding anesthetic decisions. The integration of viscoelastic testing could potentially help:

✓ Better individualize perioperative management in FXI-deficient parturients,
✓ Reduce unnecessary exposure to hemostatic agents when clot formation is functionally preserved,
✓ And support safe use of neuraxial anesthesia in selected cases with reassuring ROTEM profiles.

These hypotheses remain exploratory and underscore the need for further research. Prospective multicenter studies are warranted to evaluate whether such an approach could be formalized in future recommendations.

This case is reported in accordance with the SCARE 2025 guidelines to ensure transparency and reproducibility in surgical and anesthetic case reports.

## 4 Conclusion

This case highlights the potential value of ROTEM as a complementary tool in the anesthetic management of parturients with FXI deficiency. By providing a dynamic and functional assessment of coagulation, ROTEM may contribute to more nuanced risk stratification and support safe use of regional anesthesia in selected patients. While our findings are encouraging, they remain illustrative and hypothesis-generating. Larger prospective studies are needed to confirm the clinical utility of ROTEM in this setting and to inform future guidelines.

**Ethics statement**

Written informed consent was obtained from the patient for the publication of this case report and accompanying images.

## Author contribution

Author contributions were as follows:

▪ **Asma Ladib (Corresponding author)**: Study concept and design, supervision, writing – review and editing, final approval of the manuscript, submission, and correspondence with the journal.
▪ **Jihen Ladib**: Data analysis and interpretation.
▪ **Fethi Jebali**: Supervision, critical revision of the manuscript.
▪ **Asma Korbi**: Study concept and design, writing – original draft.
▪ **Imen Ghaddab**: Data analysis and interpretation, review and editing.
▪ **Mouna Sassi**: Supervision, critical revision of the manuscript.

## Compliance with SCARE 2025 guidelines

This case is reported in accordance with the SCARE 2025 guidelines, which standardize the reporting of surgical and anesthetic case reports to improve transparency and reproducibility.

## Ethical approval and consent statement

Written informed consent was obtained from the patient for the publication of this case report and accompanying images.

Funding statement

This research did not receive any specific grant from funding agencies in the public, commercial, or not-for-profit sectors.

## Guarantor

Asma Ladib accepts full responsibility for the work and/or the conduct of the study, had access to the data, and controlled the decision to publish.

## Declaration of Generative AI and AI-assisted technologies in the writing process

AI-assisted language editing was used under author supervision.

## Declaration of competing interest

The authors declare no conflicts of interest.
